# Inoculation of *Ensifer fredii* strain LP2/20 immobilized in agar results in growth promotion and alteration of bacterial community structure of Chinese kale planted soil

**DOI:** 10.1038/s41598-020-72986-5

**Published:** 2020-09-28

**Authors:** Neelawan Pongsilp, Pongrawee Nimnoi

**Affiliations:** 1 Department of Microbiology, Faculty of Science, Silpakorn University, Nakhon Pathom, 7300 Thailand; 2 Department of Microbiology, Faculty of Liberal Arts and Science, Kasetsart University, Nakhon Pathom, 73140 Thailand

**Keywords:** Ecology, Microbiology, Plant sciences

## Abstract

In our former research, we succeeded in using agar, alginate, and perlite as immobilization materials to maintain long-term survival of the inoculant, *Ensifer fredii* LP2/20, in a controlled glasshouse. Therefore the information on the establishment and activity of the inoculant to promote plant growth under field conditions, the effects of the inoculant on the soil microbial communities and specific microbial taxa, and the association between the inoculant and soil elements merit further studies. Here, we found that agar was the most suitable material that supported the establishment of the inoculant under field conditions. RNA-based analysis showed that *E. fredii* LP2/20 immobilized in agar was still metabolically active at day 50 after being introduced into soil. Inoculation of *E. fredii* LP2/20 immobilized in agar conferred the highest plant dry weight (up to 89.94%) and all plant elements including total N (9.55%), P (17.94%), K (68.42%), Ca (39.77%), Mg (30.76%), Fe (29.85%), and Zn (22.44%). Inoculation of *E. fredii* LP2/20 immobilized in agar increased soil chemicals including soil organic matter (99.02%), total N (272.48%), P (31.75%), K (52.74%), Fe (51.06%), and Zn (63.10%). High-throughput next-generation sequencing of bacterial 16S rRNA amplicons showed that the *Proteobacteria*, *Acidobacteria*, *Bacteroidetes*, and *Firmicutes* were dominant phyla in Chinese kale field soil. Inoculation of *E. fredii* LP2/20 significantly affected the soil bacterial community structure by decreasing total bacterial richness and diversity. The numbers of alpha*-* and gamma*-Proteobacteria* were significantly increased while the number of delta-*Proteobacteria* was significantly decreased due to *E. fredii* LP2/20 establishment. Soil total P, K, and Ca and soil pH were the important factors that shaped the soil bacterial community composition.

## Introduction

Nowadays, modern agricultural practices focus on the use of biofertilizers to move towards agricultural and environmental sustainability. One approach to reach the goal is to abandon the use of chemical fertilizers, herbicides, and pesticides because they have enhanced a range of environmental problems such as reduction in soil quality and biodiversity^1^. The use of green fertilizers or biofertilizers instead develops an eco-friendly application.

Biofertilizers are preselected effective microorganisms that play important roles in regulating soil fertility, nutrient cycling, and promoting plant growth. Bacteria that confer many advantages to plants and are potentially useful for plant growth stimulation are termed as plant growth-promoting bacteria (PGPB). The major groups of PGPB belong to the alpha-, beta-, and gamma-*Proteobacteria*, *Actinobacteria*, *Bacteroidetes* as well as *Firmicutes*^1,2^. Using PGPB as biofertilizers provides multiple benefits in agriculture by enhancing crop productivity and plant nutrient content and suppressing plant pathogens^1,3,4^. PGPB directly facilitate plant growth by improving the uptake of macronutrients such as nitrogen (N), phosphorus (P), potassium (K), calcium (Ca), and magnesium (Mg) as well as micronutrients such as iron (Fe), zinc (Zn), and copper (Cu)^5,6^. A decrease in soil pH resulting from the production of organic acids or the stimulation of proton-pumping ATPase improves the solubilization of those nutrients^7^. However, there are some problems concerning the colonization and persistence of the inoculant after its introduction into the competitive soil environment such as the attack by bacteriophage or protozoa^8^.

Our former research had been conducted to determine the potential of using agar and alginate compared with perlite, which is currently used in biofertilizer production, as cell immobilization materials for maintaining long-term survival of the inoculants strain in a controlled glasshouse and reducing the production cost. Soil bacteria were isolated from an organic farm in Lopburi province of Thailand and evaluated for their abilities to promote plant growth. In a former research we succeeded in using agar, alginate, and perlite as cell immobilization materials to maintain long-term survival of *E. fredii* LP2/20 that produced high amounts of plant stimulants including indole acetic acid (IAA) (42.71 ± 0.85 µg/ml) and ammonia (0.79 ± 0.01 mM) in a controlled glasshouse. *E. fredii* LP2/20 immobilized in agar exhibited the highest survival (more than 10^6^ CFU/g soil) and was still metabolically active throughout the cultivation period. Moreover, morphological observation of the immobilized cells, performed under a scanning electron microscope (SEM), revealed that *E. fredii* LP2/20 cells were rod-shaped and present in the forms of single cells and microcolonies producing extracellular polymeric materials on the agar surface^9^. However, the activity and establishment of *E. fredii* LP2/20 immobilized in agar, alginate, and perlite after being introduced into the competitive soil under field conditions have never been investigated. The information on the effect of *E. fredii* LP2/20 on the soil bacterial community composition, such as bacterial taxa that are stimulated by the inoculant, and soil parameters that are connected to each taxon and influence plant growth and health, is still insufficiently understood. In this study, we used *Brassica oleracea* var. *alboglabra* (Chinese kale) as the target plant because it is one of the popular leaf vegetables among Southeast Asian countries. The cost of chemical fertilizers for Chinese kale cultivation is still high, around 80–90 U.S. dollars (USD) per hectare (Office of Agricultural Economics, Thailand). The cultivated area of Chinese kale in Thailand is approximately 4.3% in proportion to the vegetable-cultivated area (4722.9 km^2^), and the use of chemical fertilizers and insecticides is still involved in many traditional cultivation approaches^10^.

We therefore conducted the experiment to examine: (1) the potential of using *E. fredii* LP2/20 immobilized in agar, alginate, and perlite to promote plant growth under field conditions, (2) the influence of *E. fredii* LP2/20 immobilized in agar, alginate, and perlite on plant nutrients and soil elements, (3) the effect of using *E. fredii* LP2/20 immobilized in agar, alginate, and perlite on the soil bacterial community structure assessed by high-throughput sequencing of 16S rRNA gene amplicons as well as (4) the changes in the abundance and associations of bacterial taxa in response to soil environmental factors. Our experiments were carried out with the expectation of moving towards an environmentally friendly manner for sustainable development.

## Material and methods

### Experimental design and soil sample collection

The experiment was conducted in the field at Pho Kao Ton subdistrict, Mueang district, Lopburi province of Thailand (latitude 14° 47′ 34.2″ N longitude 100° 36′ 13.7″ E) where Chinese kale has been regularly and consecutively cultivated twice a year from November to April for over 5 years. Two kg of each immobilization material (agar, alignate, and perlite) encapsulating *E. fredii* LP2/20 at a final concentration of 10^9^ cells/kg were prepared as described by Nimnoi et al.^9^ and then introduced into soil. The immobilization materials were sowed and cell cultures were sprayed directly over the soil by using a knapsack sprayer. The soil was mixed by shoveling over several times. Five treatments (three replicates of each treatment, 15 plots in total) were arranged in a completely randomized design. The treatments included SA (uninoculated control), SB (inoculation with *E. fredii* LP2/20 in liquid medium), SC (inoculation with *E. fredii* LP2/20 immobilized in agar), SD (inoculation with *E. fredii* LP2/20 in alginate), and SE (inoculation with *E. fredii* LP2/20 immobilized in perlite). Chinese kale seeds were planted in 1.50 m × 7.50 m plots with a 1-m interval between plots to avoid cross-contact among the different treatments. Watering was performed around 1.5 mm/day and a temperature range was between 27 and 38 °C. Fifty days after planting, Chinese kale planted soil from each individual plot were collected from a 0- to 20-cm depth with sterile soil augers according to a staggered-grid method^11^ and mixed thoroughly. The mixed soil samples were divided into two portions; one was subjected to soil physicochemical analyses and the other was subjected to soil DNA and RNA extraction. For the former portion, 6 kg of soil from each treatment were collected using sterile trowels, placed in a sterile plastic bag, and immediately stored in an ice box. For the latter portion, 20 g of soil from each treatment were preserved with RNA*later* Stabilization Solution (ThermoFisher Scientific, Waltham, MA, USA) and immediately stored in an ice box. After arrival at the laboratory, soil samples of both portions were immediately stored in a − 80 °C refrigerator.

### Soil physicochemical analyses

Two kg of soil from each plot were air-dried, sieved through a 2-mm/10 mesh, and then subjected to soil physicochemical characteristic study. The samples were analyzed for particle-size distribution following the method described by Bouyoucos^12^. Organic matter (OM) content was analyzed as described by Walkley and Black^13^. Amounts of P, Fe, Ca, and Zn were analyzed by the Bray II method^14^. Amounts of K and Mg were determined by the flame photometric method^15^. Quantity of available N was estimated as described by Li et al.^11^. pH and electrical conductivity (EC_e_) values were measured^15^.

### Plant growth promotion and plant nutrient analyses

Plant growth-promoting abilities were determined based on plant dry weight and plant nutrients including total N, P, K, Ca, Mg, Fe, and Zn. Plant materials from triplicate plots were dried in an oven for approximately 72 h at 65 °C until constant weight was obtained. Dried plant materials were ground in a mortar and digested with a mixture of H_2_SO_4_, Se, and Na_2_SO_4_ modified from the Bergersen method^16^. The total N was determined by the Kjeldahl (wet oxidation) method^17^. Quantity of P was determined spectrophotometrically according to the vanadate-molybdate procedure^18^. Amounts of K, Ca, Mg, Fe, and Zn were measured by an atomic absorption spectrophotometer (AAS; Perkin-Elmer 3100) (Perkin-Elmer, Waltham, MA, USA) according to the Association of Official Analytical Chemists (AOAC) methods^19,20^.

### Soil DNA extraction and Illumina next-generation sequencing (NGS)

Three replicates of soil samples from each treatment were subjected to total DNA extraction using PowerSoil DNA Isolation Kit (MoBio Laboratories, Carlsbad, CA, USA) according to the manufacturer’s instruction. After extraction, the purity and quantity of DNA were determined by using a DS-11FX + Spectro/Fluorometer (DeNovix, Wilmington, DE, USA). The V4 variable region of the 16S rRNA gene was amplified by using the 515F and 806R specific primer set with the barcodes^21,22^. PCR reactions were carried out with Phusion High-Fidelity PCR Master Mix (New England Biolabs, Ipswich, MA, USA). The PCR products were purified using Qiagen gel extraction kit (Qiagen, Germantown, MD, USA). The libraries were generated with TruSeq DNA PCR-Free sample preparation kit (Illumina, San Diego, CA, USA) and analyzed by HiSeq2500 PE250 sequencing system (Illumina, San Diego, CA, USA) according to the manufacturer’s instructions. Negative controls (sterile water) were carried out through amplification and sequencing. Data was returned as fastq files and deposited in the Sequence Read Archive of the National Center for Biotechnology Information under BioProject accession number:SRP154296.

### Data processing and bioinformatic analyses

Paired-end reads were merged by using the FLASH program version 1.2.7^23^ (https://ccb.jhu.edu/software/FLASH/). Quality filtering on the raw tags was performed to obtain the high-quality clean tags according to the QIIME software version 1.7.0^24^ (https://qiime.org/scripts/split_libraries_fastq.html). The tags were compared with the reference database using the UCHIME algorithm (https://www.drive5.com/usearch/manual/uchime_algo.html) to detect chimera sequences. Chimera sequences were removed to obtain the effective tags^25^. For operational taxonomic unit (OTU) clustering and species annotation, sequence analysis was performed with all effective tags by using the Uparse software version 7.0.1001^26^ (https://drive5.com/uparse/). Sequences with ≥ 97% similarity were assigned to the same OTUs. The Mothur software version 1.36.1^27^ (https://mothur.org/wiki/mothur_v.1.36.0/) was used to align each representative sequence against the SSU rRNA database of SILVA (https://www.arb-silva.de/) for species annotation at each taxonomic level^28^. The phylogenetic relationship of all OTUs derived from representative sequences was analyzed by using the MUSCLE program version 3.8.31^29^ (https://www.drive5.com/muscle/).

### Reverse transcription PCR (RT-PCR)-denaturing gradient gel electrophoresis (DGGE) analysis

The persistence and traceability of *E. fredii* LP2/20 after being introduced into soil were determined by using RT-PCR-DGGE. Fifty days after planting, three replicates of soil from each treatment were collected and subjected to total RNA extraction using RNA PowerSoil Total RNA Isolation Kit (MoBio Laboratories, Carlsbad, CA, USA) according to the manufacturer’s instruction. After extraction, the purity and quantity of total RNA were determined by using a DS-11FX + Spectro/Fluorometer (DeNovix, Wilmington, DE, USA). Total RNA extracted from soil from each treatment was used as a template to amplify 16S rRNA gene by SuperScript III One-Step RT-PCR System with Platinum*Taq* DNA Polymerase (Thermo Fisher Scientific, Waltham, MA, USA) together with the forward primer F341with GC clamp and the reverse primer R534^30^. PCR reactions and conditions were performed as described in Nimnoi et al.^3^. The PCR fragments were separated by using DGGE performed with the DCode Universal Mutation Detection System (Bio-Rad Laboratories, Hercules, CA, USA) with 8% polyacrylamide gel with a liner gradient of 15–45% denaturant. Electrophoresis was performed at 130 V for 5 h at a constant temperature of 60 °C. Gels were stained with GelStar Nucleic Acid Gel Stain (Cambrex Bio Science, Rockland, ME, USA) and visualized with a gel documentation system (Dark Hood DH-50, Biostep, Germany). The 16S rRNA bands that migrated at the same position with the 16S rRNA band of *E. fredii* LP2/20 were excised from gels and subjected to cloning by using pCR8/GW/TOPO TA Cloning Kit with One Shot Mach1-T1 *E. coli* (Thermo Fisher Scientific, Waltham, MA, USA) and sequenced at the First Base, Inc. (Selangor Darul Ehsan, Malaysia).

### Statistical analyses

Alpha diversity, including community richness (Chao1 and ACE estimators), community diversity (Shannon–Weaver and Simpson’s indices), and index of sequencing depth (the Good’ coverage) as well as rarefaction data were calculated by using the QIIME software version 1.7.0^24^ (https://qiime.org/scripts/split_libraries_fastq.html) and displayed by the R software version 2.15.3^31^ (https://www.R-project.org). Principal coordinate analysis (PCoA) was performed to visualize complex, multidimensional data, which was then displayed by the WGCNA, stat, and ggplot2 packages^32^ in the R software version 2.15.3^31^ (https://www.R-project.org). The unweighted-pair group method with arithmetic mean (UPGMA) clustering was performed as a type of hierarchical clustering method to interpret the distance matrix using average linkage and conducted by using the QIIME software version 1.7.0^24^ (https://qiime.org/scripts/split_libraries_fastq.html). The nonparametic method, analysis of similarity (ANOSIM), was conducted by the R software version 2.15.3^31^ (https://www.R-project.org) to determine whether the bacterial community structures have significant inter-group and inner-group differences. Analysis of molecular variance (AMOVA) was performed using the Mothur software version 1.36.1^27^ (https://mothur.org/wiki/mothur_v.1.36.0/). Plant dry weight, plant nutrition, soil physicochemical parameters, and alpha diversity indices were subjected to analysis of variance (ANOVA) using Tukey’s test. Spearman rank correlation was used to analyze the correlations between physicochemical parameters of environmental samples and bacterial communities^33^. ANOVA and Spearman rank correlations were performed with the SPSS statistical software version 19.0 (IBM Corp., Chicago, IL, USA). Values were considered significantly different if *P* values < 0.05.

## Results

### Effect of *E. fredii* LP2/20 on plant growth and plant nutrition

Results obtained from the study (Table 1) show that all of the immobilization treatments significantly increased plant dry weight when compared with that of liquid inoculation and uninoculated control. The SC was the best one among those immobilization treatments because it conferred significantly highest plant dry weight. When compared with the SA, the SC mostly increased plant dry weight up to 89.94%, followed by the SD and SE that significantly increased plant dry weight up to 49.73% and 42.32%, respectively, while the SB did not. Moreover, the SC also significantly increased all plant elements, except total Ca and Fe, when compared with those of other treatments. When compared to the SA, the SC mostly increased total N, P, K, Ca, Mg, Fe, and Zn up to 9.55%, 17.94%, 68.42%, 39.77%, 30.76%, 29.85%, and 22.44%, respectively, whereas the SD significantly increased total K, Mg, and Fe and the SE significantly increased only total Fe. There were no significant differences in all plant elements between the SA and SB.

**Table 1:** Plant dry weight and plant nutrition from five treatments at 50 days after planting.

Parameter	Treatment				
	SA	SB	SC	SD	SE
Dry weight (kg/plot)	1.89 ± 0.29c**	2.05 ± 0.09c	3.59 ± 0.14a	2.83 ± 0.19b	2.69 ± 0.18b
Total N (%)*	4.08 ± 0.04b	4.08 ± 0.04b	4.47 ± 0.09a	4.24 ± 0.05b	4.21 ± 0.08b
Total P (%)*	0.39 ± 0.01b	0.40 ± 0.00b	0.46 ± 0.00a	0.41 ± 0.00b	0.40 ± 0.00b
Total K (%)*	0.95 ± 0.04c	0.98 ± 0.04c	1.60 ± 0.02a	1.26 ± 0.03b	0.98 ± 0.05c
Total Ca (%)*	1.81 ± 0.08b	2.10 ± 0.11ab	2.53 ± 0.30a	2.23 ± 0.14ab	2.26 ± 0.10ab
Total Mg (%)*	0.39 ± 0.00c	0.41 ± 0.00bc	0.51 ± 0.01a	0.43 ± 0.01b	0.42 ± 0.02bc
Total Fe (mg/kg)*	247.91 ± 15.18c	260.23 ± 23.37bc	321.93 ± 6.97a	301.24 ± 7.70ab	285.29 ± 8.61ab
Total Zn (mg/kg)*	30.07 ± 0.88b	30.86 ± 1.34b	36.82 ± 2.06a	32.59 ± 0.56b	31.54 ± 0.39b

Treatment SA, uninoculated control; SB, inoculation with *E. fredii* LP2/20 in liquid medium; SC, inoculation with *E. fredii* LP2/20 immobilized in agar; SD, inoculation with *E. fredii* LP2/20 immobilized in alginate; SE, inoculation with *E. fredii* LP2/20 immobilized in perlite.

*All values are the means from three samplings ± standard deviations.

**Values with the same letters within a row are not significantly different according to Tukey’s test.

### Change in soil physicochemical characteristics

The physicochemical characteristics of soil from each treatment at 50 days after planting were investigated. The results (Table 2) show that all of the immobilization treatments significantly increased soil OM and four soil elements including total K, Ca, Fe, and Zn when compared with those of liquid inoculation and uninoculated control. The SC was the only treatment able to significantly increased all of soil elements when compared with those of liquid inoculation and uninoculated control. The SC resulted in the significantly highest amounts of soil OM, total N, and P. The SC and SE resulted in the significantly highest amounts of total K and Zn. The SC and SD resulted in the significantly highest amount of total Fe. The SD and SE resulted in the highest level of total Ca which was significantly higher than that of liquid inoculation and uninoculated control. The SC and SE resulted in the highest level of total Mg which was significantly higher than that of liquid inoculation and uninoculated control. The SC exhibited the highest increases in OM (99.02%), total N (272.48%), P (31.75%), K (52.74%), Fe (51.06%), and Zn (63.10%), as compared to uninoculated control. The SE exhibited the highest increases in total Ca and Mg up to 62.36% and 12.36%, respectively, as compared to uninoculated control. In addition, soil samples from all treatments were categorized into silty clay loam having pH values of 7.84 to 8.01 and similar in soil physical property in terms of percentages of sand, silt, and clay.

**Table 2:** Physicochemical properties of soil from five treatments at 50 days after planting.

Parameter	Treatment				
	SA	SB	SC	SD	SE
pH	8.01	7.89	7.88	7.96	7.84
Electrical conductivity (ds/m)	1.51	1.74	2.04	1.94	1.70
Organic matter (%)*	1.03 ± 0.03c**	1.06 ± 0.01c	2.05 ± 0.05a	1.68 ± 0.01b	1.60 ± 0.13b
Total N (mg/kg)*	29.08 ± 1.14d	66.37 ± 4.05c	108.32 ± 4.72a	100.26 ± 1.36b	71.56 ± 1.42c
Total P (mg/kg)*	75.51 ± 0.71d	79.97 ± 0.18c	99.49 ± 0.54a	86.26 ± 1.62b	79.04 ± 0.69c
Total K (mg/kg)*	49.58 ± 0.60c	52.77 ± 2.12c	75.73 ± 1.96a	70.27 ± 0.33b	74.46 ± 1.33a
Total Ca (mg/kg)*	1785.53 ± 39.80e	2018.60 ± 116.76de	2311.57 ± 49.79bc	2501.16 ± 275.68ab	2899.02 ± 141.04a
Total Mg (mg/kg)*	755.19 ± 23.45b	756.05 ± 28.98b	847.55 ± 12.59a	806.17 ± 16.10ab	848.58 ± 38.65a
Total Fe (mg/kg)*	45.57 ± 0.80c	46.73 ± 0.73c	68.84 ± 6.54a	68.37 ± 6.67a	51.69 ± 2.29b
Total Zn (mg/kg)*	10.49 ± 0.25c	10.44 ± 0.21c	17.11 ± 0.21a	13.28 ± 0.35b	17.00 ± 1.20a
Sand (%)	14.82	15.66	15.81	17.41	15.87
Silt (%)	45.53	45.57	50.54	49.59	50.50
Clay (%)	39.65	38.77	33.65	33.00	33.63
Texture	Silty clay loam	Silty clay loam	Silty clay loam	Silty clay loam	Silty clay loam

Treatment SA, uninoculated control; SB, inoculation with *E. fredii* LP2/20 in liquid medium; SC, inoculation with *E. fredii* LP2/20 immobilized in agar; SD, inoculation with *E. fredii* LP2/20 immobilized in alginate; SE, inoculation with *E. fredii* LP2/20 immobilized in perlite.

*Values are the means from three samplings ± standard deviations.

**Values with the same letters within a row are not significantly different according to Tukey’s test.

### Sequencing analysis, bacterial diversity, and richness indices

**Figure 1:**
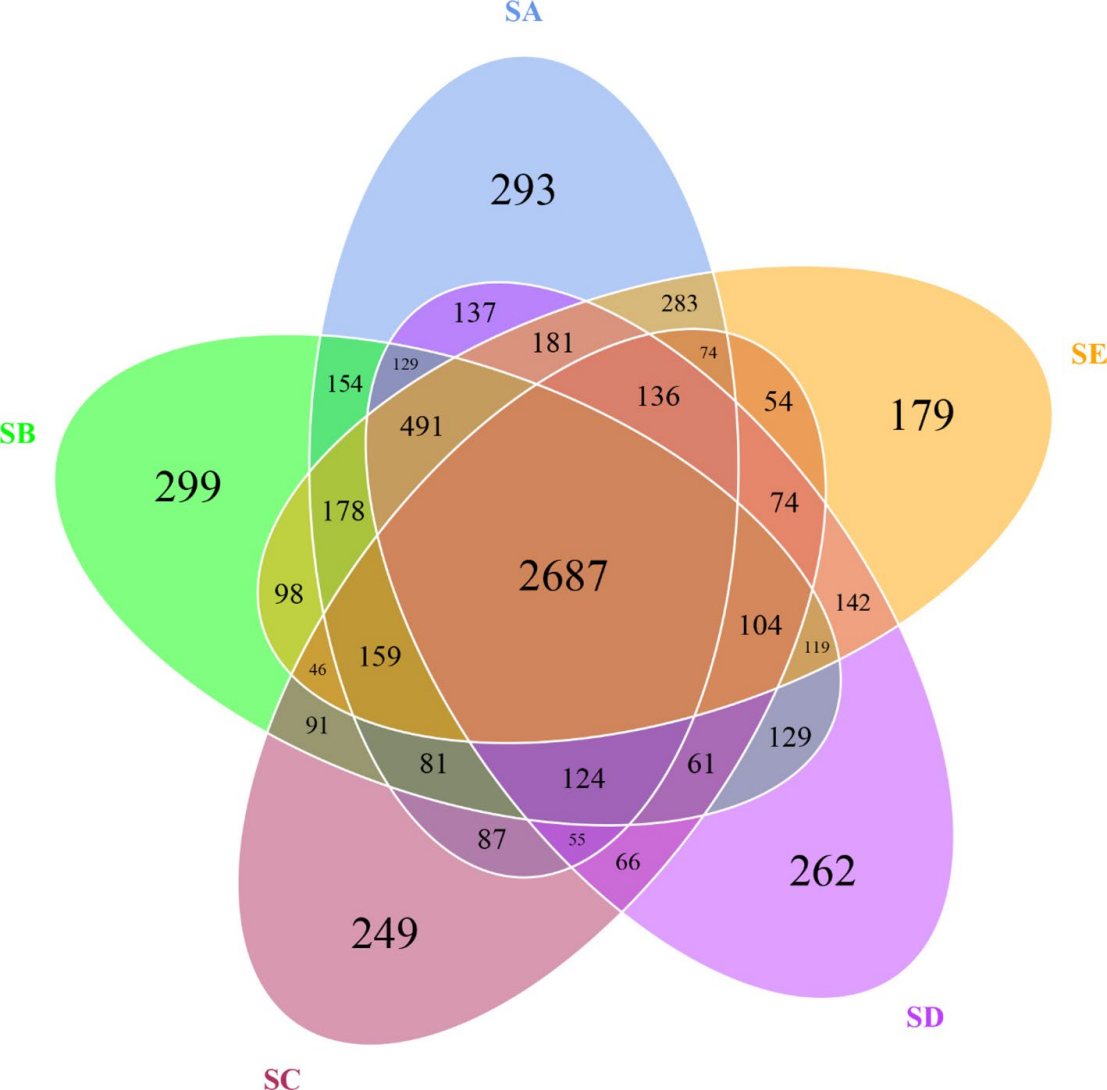
Venn diagram presenting the numbers of common, overlapping, and unique OTUs among treatments. SA, uninoculated control; SB, inoculation with *E. fredii* LP2/20 in liquid medium; SC, inoculation with *E. fredii* LP2/20 immobilized in agar; SD, inoculation with *E. fredii* LP2/20 immobilized in alginate; SE, inoculation with *E. fredii* LP2/20 immobilized in perlite.

In this study, a total of 1,198,141 raw reads were obtained from 15 DNA samples (three replicates/treatment). After tag merge and quality control, a total of 1,159,168 qualified tags (96.74% of raw reads) were obtained. Potential chimera tags were removed from qualified tags by the UCHIME algorithm and 647,864 taxon tags were remained. The tags with ≥ 97% similarity were grouped into the same OTUs. A total of 7317 OTUs were observed from all treatments, with a mean Good’s coverage of 98.00 ± 0.00%. The Venn diagram (Fig. 1) illustrates the numbers of common, overlapping, and unique OTUs among treatments. The result exhibits the common 2687 OTUs in all treatments. The SB had the highest unique OTUs (299 OTUs), followed by the SA, SD, SC, and SE, respectively. In addition, Shannon–Weaver and Simpson that indicate diversity, Chao1 and ACE (Abundance-based Coverage Estimator) that represent richness as well as the number of observed OTUs in each treatment were analyzed (Table 3). The results show that the SA, SB, and SE shared the highest rank of the observed OTU numbers which were significantly different when compared with that of the SC. The observed OTU numbers of the SC and SD were not significantly different. As higher Shannon–Weaver and Simpson indices indicate greater bacterial diversity, the SC exhibited the lowest bacterial diversity which was significantly different from that of other treatments. The SA possessed the highest bacterial diversity which was not significantly different from that of the SB, SD, and SE. The bacterial richness indices (Chao1 and ACE) of the SA and SC were highest and lowest, respectively, which were significantly different from each other. Both bacterial richness indices of remaining treatments were not significantly different from those of the SA and SC. The results demonstrate that inoculation with *E. fredii* LP2/20 immobilized in agar was the only treatment that significantly decreased bacterial diversity and richness.

**Table 3:** Indices of bacterial diversity and richness of soil from five treatments at 50 days after planting.

Treatment	Observed OTU*	Indices of diversity*		Indices of richness*	
		Shannon–Weaver	Simpson	Chao1	ACE
SA	3533.00 ± 249.92a**	10.04 ± 0.13a	0.99 ± 0.00a	4001.08 ± 281.81a	4087.70 ± 296.54a
SB	3347.66 ± 149.08a	9.83 ± 0.14a	0.99 ± 0.00a	3805.65 ± 168.80ab	3882.31 ± 186.38ab
SC	2657.00 ± 101.68b	8.61 ± 0.16b	0.98 ± 0.00b	3107.96 ± 62.30b	3196.83 ± 89.77b
SD	3327.00 ± 392.90ab	9.82 ± 0.34a	0.99 ± 0.00a	3762.41 ± 426.20ab	3872.47 ± 448.63ab
SE	3350.66 ± 280.32a	9.08 ± 0.26a	0.99 ± 0.00a	3789.72 ± 295.82ab	3868.19 ± 313.79ab

Treatment SA, uninoculated control; SB, inoculation with *E. fredii* LP2/20 in liquid medium; SC, inoculation with *E. fredii* LP2/20 immobilized in agar; SD, inoculation with *E. fredii* LP2/20 immobilized in alginate; SE, inoculation with *E. fredii* LP2/20 immobilized in perlite.

*Values are the means from three samplings ± standard deviations.

**Values with the same letters within a column are not significantly different according to Tukey’s test.

### Illumina NGS and bacterial community structures

**Figure 2:**
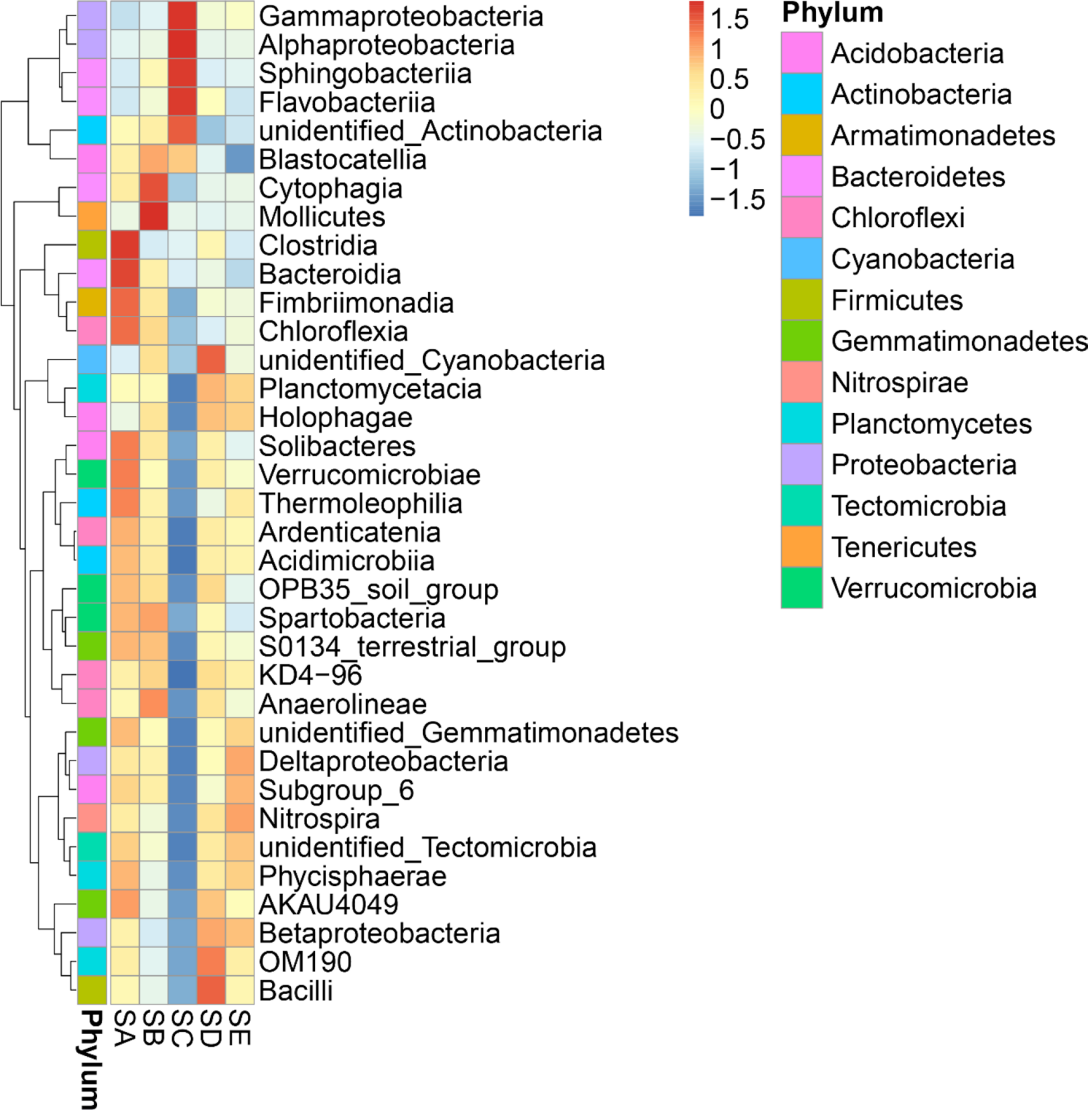
Heat map analysis of class distribution in each treatment. SA, uninoculated control; SB, inoculation with *E. fredii* LP2/20 in liquid medium; SC, inoculation with *E. fredii* LP2/20 immobilized in agar; SD, inoculation with *E. fredii* LP2/20 immobilized in alginate; SE, inoculation with *E. fredii* LP2/20 immobilized in perlite.

The phylum *Proteobacteria* was mostly abundant in all treatments, ranging between 35.87 and 53.52%, followed by the *Acidobacteria* (9.87–13.21%), *Bacteroidetes* (10.33–12.98%), *Firmicutes* (5.73–6.73%), *Cyanobacteria* (1.21–6.21%), *Gemmatimonadetes* (3.23–5.96%), *Chloroflexi* (2.69–5.50%), *Planctomycetes* (1.96–4.82%), *Actinobacteria* (3.21–4.33%), and *Thaumarchaeota* (1.41–3.07%). The distribution of bacterial classes in each treatment is shown in Fig. 2. The colors in a heat map chart indicate the abundance distribution of the community. The colors which vary from deep blue to dark brown represent low- to high-levels of the relative abundance. The most abundant classes in each site are represented as dark-brown squares in a heat map chart. In the SA, the *Clostridia*, *Bacteroidia*, *Fimbriimonadia*, and *Chloroflexia* were more abundant than others. The *Mollicutes*, *Cytophagia*, *Blastocatellia*, and *Anaerolineae* were the predominant classes in the SB. The alpha-, gamma-*Proteobacteria*, *Sphingobacteriia*, *Flavobacteriia*, and unidentified *Actinobacteria* were the predominant classes in the SC. The SD had a few most-abundant classes including the unidentified *Cyanobacteria*, *Planctomycetota* (OM190), and *Bacilli*. The SE harbored the high numbers of delta-*Proteobacteria* and *Nitrospira*.

**Figure 3:**
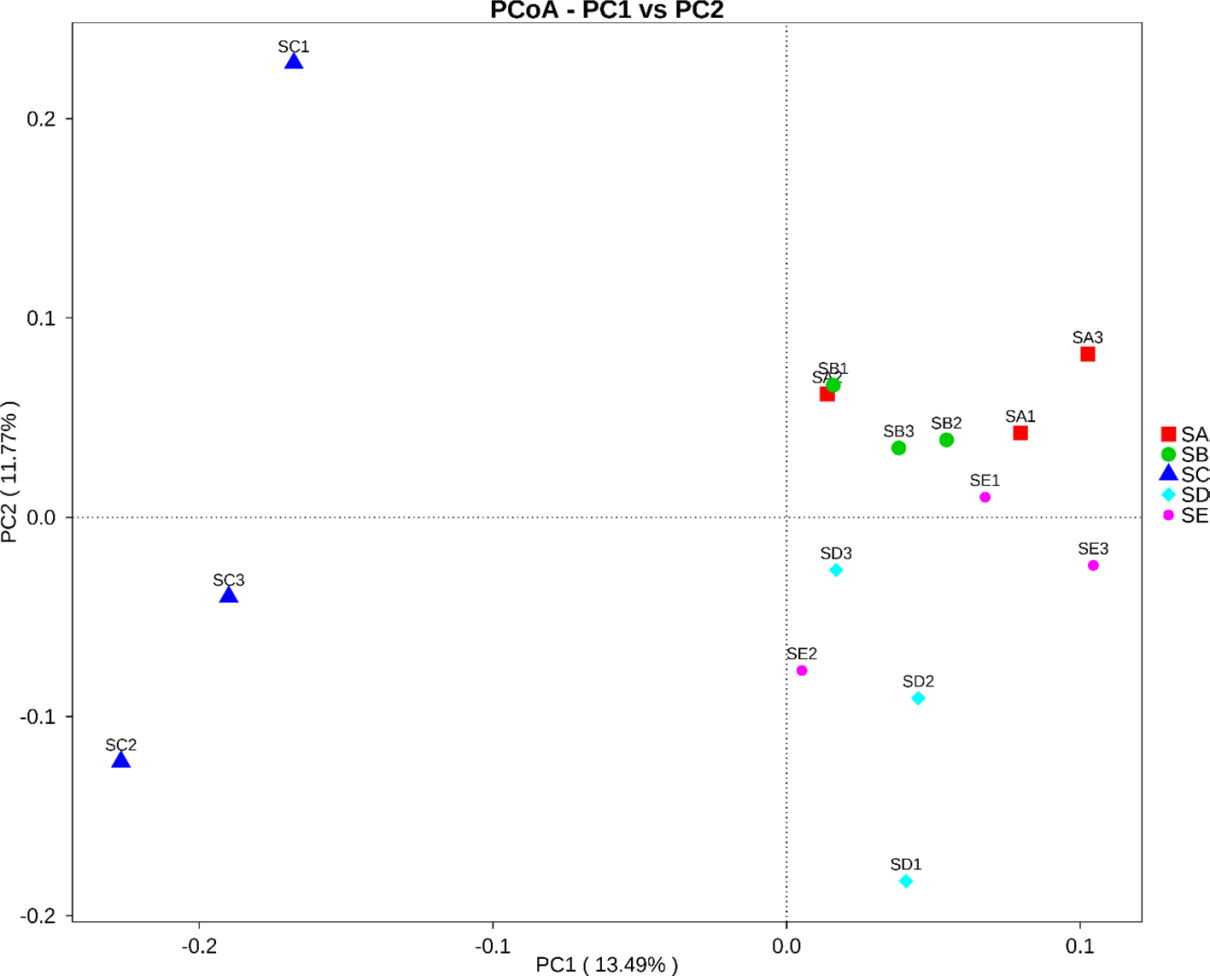
Principal Coordinate Analysis (PCoA) of bacterial composition similarity of each treatment. SA, uninoculated control; SB, inoculation with *E. fredii* LP2/20 in liquid medium; SC, inoculation with *E. fredii* LP2/20 immobilized in agar; SD, inoculation with *E. fredii* LP2/20 immobilized in alginate; SE, inoculation with *E. fredii* LP2/20 immobilized in perlite.

**Figure 4:**
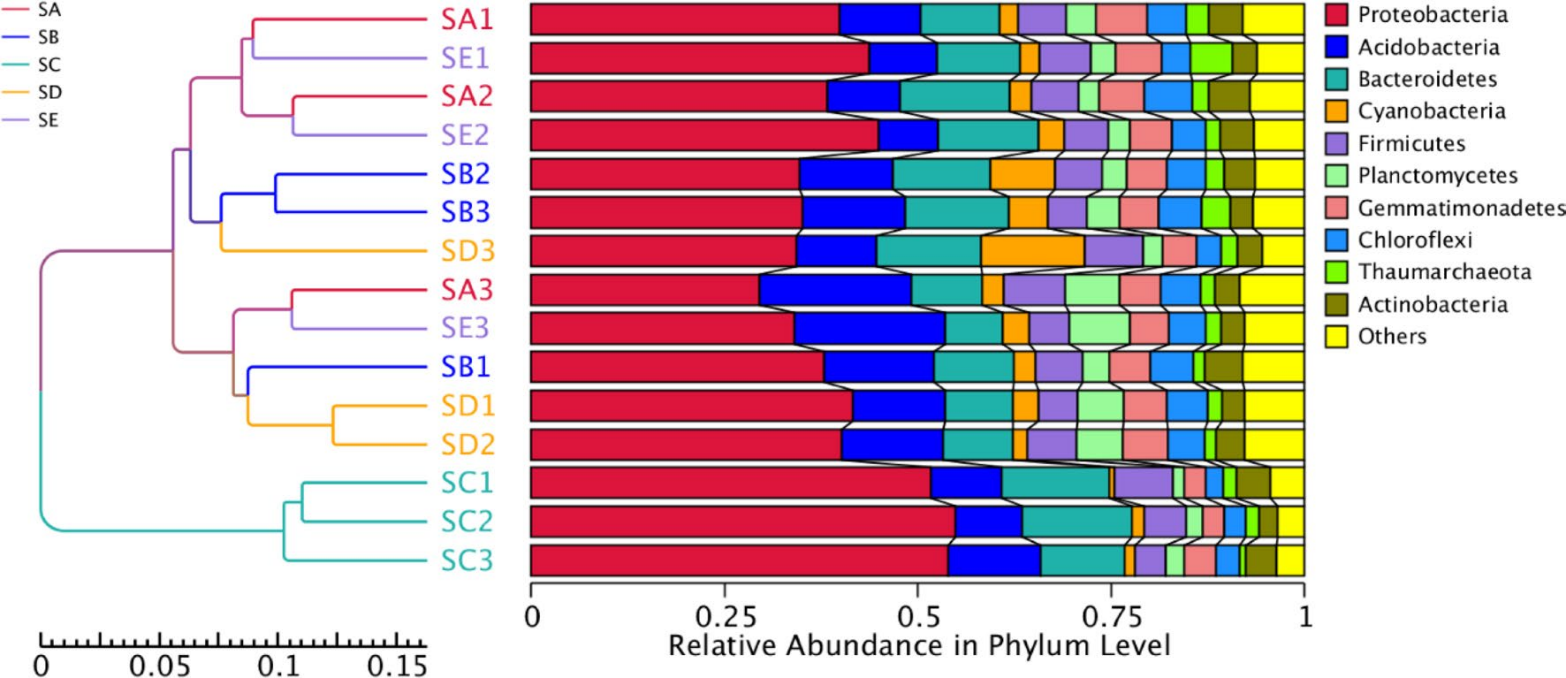
The UPGMA dendrogram of relative abundance at phylum level from three samplings of each treatment. SA, uninoculated control; SB, inoculation with *E. fredii* LP2/20 in liquid medium; SC, inoculation with *E. fredii* LP2/20 immobilized in agar; SD, inoculation with *E. fredii* LP2/20 immobilized in alginate; SE, inoculation with *E. fredii* LP2/20 immobilized in perlite.

The ordination of samples from each treatment by PCoA shown in Fig. 3 exhibited that the bacterial communities of the SC formed a separate cluster from those of other treatments. AMOVA indicated that the bacterial community structures of different treatments were significant from each other (*P* < 0.05). In addition, the variations in the inter-group and inner-group bacterial community compositions were evaluated by ANOSIM. The variations of the inter-group bacterial communities of the SC were considered significant (*P* < 0.05) from the other treatments and were larger than those of the inner-group (*R* = 1). On the contrary, the variations of the inner-group bacterial communities of the remaining treatments were not significantly different (*P* > 0.05) and were larger than those of the inter-group (*R* = −0). The UPGMA dendrogram of the relative abundance at the phylum level depicted in Fig. 4 was divided into two main clusters. The first main cluster contained only triplicate samples of the SC. The second main cluster contained samples from the remaining treatments, in which the SA and SE were closer to each other as well as the SB and SD were closer to each other. The dendrogram confirmed that inoculation of *E. fredii* LP2/20 immobilized in agar altered the soil bacterial community structure, whereas inoculations of *E. fredii* LP2/20 in liquid medium, alginate, and perlite did not affect the soil bacterial community structure. This finding may result from the persistence and cell survival of *E. fredii* LP2/20 immobilized in agar. Agar was proved to be the most suitable immobilization material that prolonged cell survival and supported the establishment of *E. fredii* LP2/20 after being introduced into soil rather than other immobilization materials and liquid medium. Thus, we further determined the establishment and activity of *E. fredii* LP2/20 in each treatment at day 50 after being introduced into soil by using RNA based techniques.

### RT-PCR-DGGE analysis

**Figure 5:**
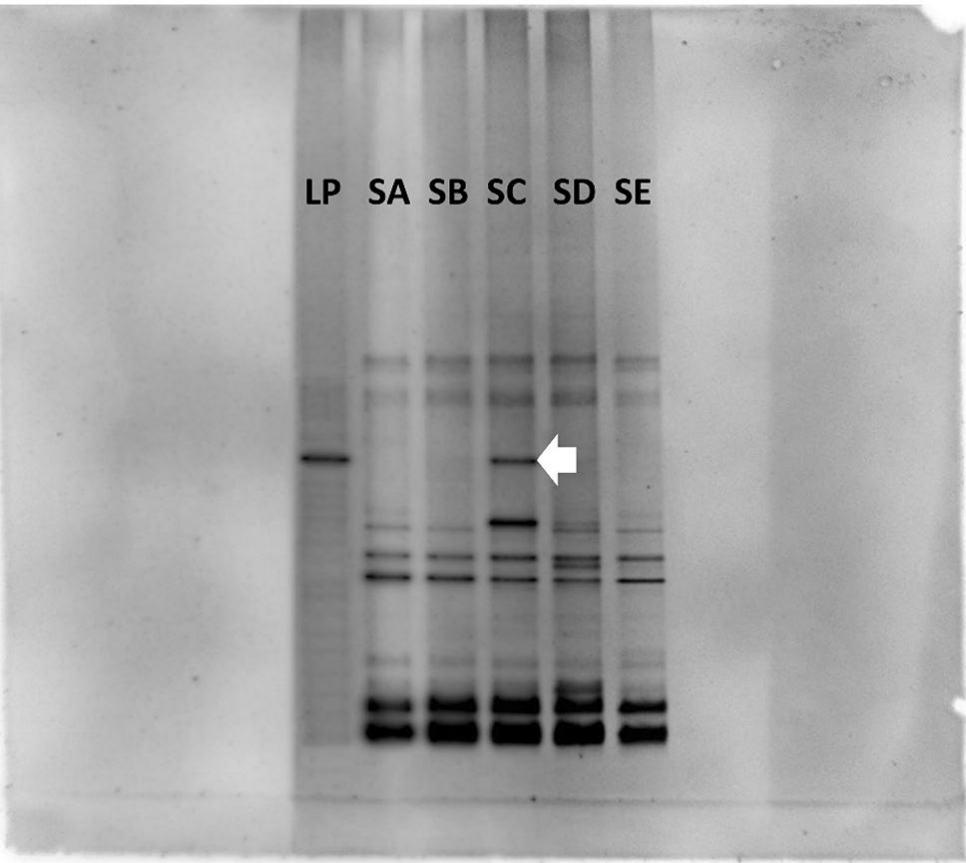
RT-PCR-DGGE fingerprintings of soil from five treatments at 50 days after planting. LP, reference band of *E. fredii* LP2/20; SA, uninoculated control; SB, inoculation with *E. fredii* LP2/20 in liquid medium; SC, inoculation with *E. fredii* LP2/20 immobilized in agar; SD, inoculation with *E. fredii* LP2/20 immobilized in alginate; SE, inoculation with *E. fredii* LP2/20 immobilized in perlite. An arrow indicates the 16S rRNA band that migrated to the same position as the reference band and was subjected to excision, cloning, and sequencing. (The original figures without labels are shown in Supplementary Figs. S1–S3).

The establishment and activity of *E. fredii* LP2/20 at day 50 after inoculation into soil were determined by using RT-PCR-DGGE. In DGGE analysis, the DGGE patterns among three replicates of each treatment were compared first and found to be identical (see Supplementary Fig. S1–S3). The idea of DGGE is employed for the separation of the fragments that are identical in length, but different in sequence. Fragments that are identical both in length and sequence possess the same migration distance in DGGE gel. As shown in Fig. 5, the 16S rRNA gene of *E. fredii* LP2/20 was used as the reference band (LP) for verification of the species-specific migration position on DGGE gel. DGGE profiles from each treatment were primarily identified by comparing their relative migration positions on gels with that of the reference band. RNA-based DGGE profiles revealed the difference in metabolically active bacteria in soil. Only the SC possessed the 16S rRNA band that migrated to the same position as the reference band, indicating that only *E. fredii* LP2/20 immobilized in agar was metabolically active on the 50th day of inoculation. To confirm this finding, the band from the SC that migrated the same distance as the reference band in DGGE gel was excised and subjected to cloning and sequencing. Sequence analysis of the cloned DGGE band confirmed that it was derived from *E. fredii* LP2/20. The sequence of the cloned band has been deposited in the GenBank database (accession number: KU866444). Thus, only *E. fredii* LP2/20 immobilized in agar could persist, remain metabolically active, and affect the soil bacterial community structure, while *E. fredii* LP2/20 in other treatments didn’t. This finding corresponds to the results from PCoA, AMOVA, ANOSIM, and UPGMA dendrogram which demonstrated that *E. fredii* LP2/20 immobilized in agar caused a significant alteration in the soil bacterial community structure.

### Effect of environmental factors and *E .fredii* LP2/20 on the distribution of soil bacterial communities

The effect of soil physicochemical parameters and *E. fredii* LP2/20 on the bacterial communities were analyzed. The results exhibit that members of the alpha-*Proteobacteria* and *Sphingobacteriia* were most positively associated with total P (Spearman’s *r* = 0.70, *P* = 0.18; *r* = 0.70, *P* = 0.18) and K (*r* = 0.70, *P* = 0.18; *r* = 0.70, *P* = 0.18) and negatively associated with pH (*r* = −0.70, *P* = 0.18; *r* = −0.70, *P* = 0.18). The gamma-*Proteobacteria* were also most positively associated with total K (*r* = 1.00, *P* = 0.00) and negatively associated with pH (*r* = −0.80, *P* = 0.10). The members of beta-, delta-*Proteobacteria*, *Acidobacteria* subgroup 6, and soil *Crenarchaeota* group (SCG) were most positively associated with total Ca (*r* = 0.50, *P* = 0.39; *r* = 1.00, *P* = 0.87; *r* = 1.00, *P* = 0.87; *r* = 0.20, *P* = 0.74) and negatively associated with total P (*r* = −0.30, *P* = 0.62; *r* = −0.90, *P* = 0.03; *r* = −0.90, *P* = 0.03; *r* = −0.70, *P* = 0.18). On the contrary, the *Blastocatellia* were most positively associated with total P (*r* = 0.50, *P* = 0.39) and negatively associated with total Ca (*r* = −0.60, *P* = 0.28). The *Cyanobacteria* and *Bacilli* were positively associated with total Ca (*r* = 0.30, *P* = 0.62; *r* = 0.38, *P* = 0.61) and pH (*r* = 0.20, *P* = 0.74; *r* = 0.41, *P* = 0.49) and negatively associated with total K (*r* = −0.30, *P* = 0.62; *r* = −0.35, *P* = 0.55). In addition, when comparing the top ten most abundant bacterial classes distributed in soil of each treatment (Table 4), the SC significantly increased the amounts of alpha- and gamma-*Proteobacteria* when compared with those of other treatments. The number of delta-*Proteobacteria* of the SC was lowest and significantly different from that of other treatments. The numbers of unidentified *Cyanobacteria*, *Sphingobacteriia*, *Acidobacteria* subgroup 6, *Blastocatellia*, *Bacilli*, and SCG of the SC were not significantly different from those of other treatments. The amounts of the top ten most abundant bacterial classes of all treatments except the SC were not significantly different from each other. These results indicate that soil total P, K, and Ca contents and soil pH were the important factors that shaped the bacterial communities. Inoculation of *E. fredii* LP2/20 immobilized in agar affected the bacterial communities and diversity by significantly increasing the amounts of alpha- and gamma-*Proteobacteria* and significantly decreasing the amount of delta-*Proteobacteria*.

**Table 4:** Top ten most abundant bacterial classes present in each treatment.

Treatment*	Class									
	alpha*-Proteobacteria*	gamma*-Proteobacteria*	Unidentified *Cyanobacteria*	*Sphingobacteriia*	beta-*Proteobacteria*	*Acidobacteria*Subgroup 6	delta*-Proteobacteria*	*Blastocatellia*	*Bacilli*	SCG
SA	10.22 ± 1.98b**	8.68 ± 0.78b	1.78 ± 0.30a	6.66 ± 2.75a	8.93 ± 1.92ab	5.15 ± 3.66a	7.77 ± 1.05a	4.74 ± 2.25a	4.91 ± 1.03a	2.24 ± 0.58a
SB	11.33 ± 0.64b	9.47 ± 1.19b	3.80 ± 1.89a	7.59 ± 1.80a	7.47 ± 0.62ab	4.77 ± 0.73a	7.43 ± 0.54a	5.29 ± 0.06a	4.83 ± 0.50a	2.47 ± 1.15a
SC	27.05 ± 0.64a	16.35 ± 1.45a	1.03 ± 0.57a	9.47 ± 1.75a	6.23 ± 0.64b	2.75 ± 0.79a	3.76 ± 0.31b	5.08 ± 0.90a	4.72 ± 1.03a	1.41 ± 0.55a
SD	10.74 ± 0.59b	10.43 ± 3.13b	5.38 ± 5.63a	6.70 ± 2.54a	10.25 ± 0.31a	4.28 ± 0.84a	7.09 ± 1.43a	4.12 ± 0.36a	5.08 ± 1.17a	1.86 ± 0.34a
SE	11.05 ± 1.96b	10.83 ± 1.48b	2.36 ± 0.58a	6.81 ± 2.28a	9.90 ± 1.08a	5.36 ± 4.36a	8.88 ± 1.53a	3.40 ± 2.21a	4.91 ± 0.56a	3.04 ± 2.06a

Treatment SA, uninoculated control; SB, inoculation with *E. fredii* LP2/20 in liquid medium; SC, inoculation with *E. fredii* LP2/20 immobilized in agar; SD, inoculation with *E. fredii* LP2/20 immobilized in alginate; SE, inoculation with *E. fredii* LP2/20 immobilized in perlite.

*All values are the means from three samplings ± standard deviations.

**Values with the same letters within a column are not significantly different according to Tukey’s test.

## Discussion

PGPB promote plant growth in many ways such as facilitating resource acquisition, modulating plant hormone levels, solubilizing nutrients, and inhibiting various pathogens which hamper the growth and development of plants^2,34^. However, the production of biofertilizer by using free bacterial cells has many disadvantages associated with the viability and stability of PGPB during the production process and storage^35^. Free PGPB cells that are directly inoculated into soil may not effectively colonize plant roots because they are susceptible to variable environments^36^. Previous publications have reported the success of using PGPB to promote plant growth. For examples, García et al.^37^ succeeded in using *E. fredii*, *Pseudomonas fluorescens*, *Chryseobacterium balustinum*, and *Serratia fonticola* to promote the growth of soybean (*Glycine max*) under controlled glasshouse conditions. Karlidag et al.^5^ used *Bacillus* and *Microbacterium* as biofertilizers to increase yield and leaf P, K, Fe, Mn, Cu, and Zn contents of apple (*Malus domestica* L.). Turan et al.^6^ also conducted the experiments to investigate the effect of *Bacillus megaterium*, *Bacillus subtilis*, and *Pantoea agglomerans* on the growth of cabbage (*Brassica oleracea*) in a glasshouse. Many publications have reported the ability of *E. fredii* as PGPB by facilitating resource acquisition to provide plants with resources and nutrients that are unavailable via the mechanisms such as nitrogen fixation, ammonification, phosphorus and iron uptake, as well as modulation of phytohormones such as IAA that stimulates division, extension, and differentiation of plant cells^37,38^. Jiménez-Guerrero et al*.*^39^ succeeded in using *E. fredii* HH103 to increase nodulation and plant dry weight of *G. max*. Temprano-Vera et al*.*^40^ used *E. fredii* HH103 and NGR234 to promote the growth of *G. max* and *Glycine soja* and found that the strain HH103 significantly increased shoot dry weight of *G. max* and *G. soja* up to five- and four-fold, respectively when compared with uninoculated control. The strain NGR234 also significantly increased shoot dry weight of *G. max* and *G. soja* up to 0.5- and 2.5-fold, respectively when compared with uninoculated control. However, there are limited reports about the use of PGPB to promote plant growth under filed conditions, the traceability and activity of the inoculant as well as the impact of the inoculant on the microbial community under natural conditions that was analyzed by high-throughput sequencing to provide new insights into soil microbial community and diversity. Thus, in this study, we evaluated the potential of using agar, alginate, and perlite as cell immobilization materials to prolong survival of the inoculant and promote plant growth under field conditions. The results show that *E. fredii* LP2/20 immobilized in agar was the best approach that mostly increased all plant growth parameters including plant dry weight (up to 89.94%) and all plant elements (total N, P, K, Ca, Mg, Fe, and Zn) which were significantly different from those of uninoculated control. When compared to uninoculated control, inoculation of *E. fredii* LP2/20 in alginate significantly increased plant dry weight, total K, Mg, and Fe and inoculation of *E. fredii* LP2/20 immobilized in perlite significantly increased only plant dry weight and total Fe, whereas inoculation of *E. fredii* LP2/20 in liquid medium failed to promote plant growth in all parameters.

Perlite and peat have been used as carrier materials to prevent bacterial cells from soil competitive conditions^41,42^. However, there are shortages of natural peat and perlite in some countries or the peat- and perlite-mines are located in forbidden areas^43^. Due to these limitations, alternative immobilization materials such as alginate have been evaluated^44^. Alginate is the most common polymer for the encapsulation of bacteria for various industrial^45,46^ and agricultural purposes^47,48^. A few studies on the effect of alginate-encapsulated bacteria on the cotton (*Gossypium hirsutum* L.) growth and bacterial community under normal and salinity stress conditions have been reported^49,50^. However, alginate-encapsulated inoculants are more expensive than peat-based inoculants and require more complicated technical handling^9^. Therefore the use of agar as an immobilization material has been proposed to offer advantages over the other immobilization materials such as cheaper cost, sufficient availability, and friendliness towards environments. Minaxi^51^ and Jain et al.^52,53^ used agar as an immobilization material for phosphate-solubilizing bacterial and fungal strains. The experiments showed that cells immobilized in agar had significantly higher phosphate solubilization than did free cells and cells immobilized in other materials. Kiran et al.^45^ and Sankaralingam et al.^54^ reported that agar was the most effective and suitable matrix to immobilize bacterial cells, with the advantages of a higher enzyme activity, greater resistance to environmental perturbations, and a lower production cost.

Our results agree with Schoebitz et al.^35^ who reported that peat, perlite, and clay had high variability in chemical composition which affected the stability and survival of microorganisms and decreased the shelf life of the inoculants. Even though liquid inoculation simplifies inoculant production and application for the farmers, bacterial survival is decreased because there is no cell protective agent against environmental stresses. In this study we also found that all of immobilization treatments significantly increased all soil elements, except total Mg when compared with those of uninoculated control. The SC led to the highest increases of soil OM, total N, P, K, Fe, and Zn. These increases could result from the establishment, proliferation, and metabolic activity of *E. fredii* LP2/20. Agar was the best immobilization material that prolonged survival and supported its establishment in soil competitive environments. Soil is complex and dynamic in which its biological activity is mostly governed by dominant bacterial species. The dominant bacterial genera such as *Bacillus*, *Pseudomonas*, *Rhizobium*, and *Azospirillum* influence soil microenvironments and play roles in soil biogeochemical cycling and ecological processes which confer beneficial effects on soil and crop productivity^1,11^.

The 16S rRNA amplicon deep sequencing showed that the bacterial diversity across all treatments was dominated by the phyla *Proteobacteria*, *Acidobacteria*, *Bacteroidetes*, and *Firmicutes*. Our result corresponds to those in previous studies with agricultural soils^55,56^. The bacterial community of the SC formed a well-separated cluster from that of other treatments. Inoculation of *E. fredii* LP2/20 immobilized in agar also affected the bacterial communities and diversity by significantly increasing the amounts of alpha- and gamma-*Proteobacteria* and significantly decreasing the amount of delta-*Proteobacteria*. To understand the reason why only the SC significantly impacted the soil bacterial communities and diversity, while the other treatments didn’t, RT-PCR-DGGE was employed to investigate the establishment and activity of *E. fredii* LP2/20 in soil. The result from RNA-based analysis revealed that agar was the only one among immobilization materials that was capable of preserving and maintaining *E. fredii* LP2/20 alive throughout the 50-day cultivation period. On the contrary, alginate and perlite were less suitable immobilization materials under field conditions. This finding still corresponds to our former report that exhibited the superior ability of agar in maintaining cell survival of the inoculant^9^. Agar is a natural polymeric hydrogel extracted from seaweed that protects cells from stresses, desiccation, and environmental factors^35^. This study also showed that immobilization in agar maintained long-term cell survival, leading to promotion of metabolic activity of the inoculant and effects on the soil bacterial community and diversity. Usually PGPB inoculants affect the soil bacterial communities and diversity after their establishment by both direct and indirect mechanisms such as digestion of soil composition to release ammonia, phosphorus, and iron, modulation of the effects of environmental stresses, modulation of phytohormones such as IAA that stimulates plant root development, and production of exudates which subsequently affect soil bacteria^38^. *E. fredii* LP2/20 has been reported to produce extracellular polysaccharides (EPS)^9^ containing many carbon and nitrogen compounds such as ammonium sulfate, potassium nitrate, glucose, and mannose that affected other soil bacteria and subsequently affected soil nutrient recycling^57^. In addition, the relations between soil physicochemical properties and bacterial community were addressed. Soil total P, K, and Ca and soil pH were the dominant factors influencing the soil bacterial community structure. This result agrees with the reports of Li et al.^11^, O’Brien et al.^58^, and Zhang et al*.*^59^ which showed that available nutrient, P, and pH were ones of important factors affecting the soil bacterial community. The pH value and soil N, P, and K contents were correlated with the soil microbial composition^60^.

## Conclusions

Our results show the new potential use of *E. fredii* LP2/20 immobilized in agar as a bacterial biofertilizer to promote plant growth and increase plant nutrition and soil fertility in the field. Inoculation of *E. fredii* LP2/20 immobilized in agar significantly increased the highest plant dry weight up to 1.89-fold over uninoculated control, followed by *E. fredii* LP2/20 immobilized in alginate (1.49-fold) and perlite (1.42-fold), respectively. In addition, inoculation of *E. fredii* LP2/20 immobilized in agar also mostly increased all plant elements including total N, P, K, Ca, Mg, Fe, and Zn and altered soil chemicals by increasing soil OM, total N, P, K, Fe, and Zn. High-throughput sequencing of 16S rRNA gene exhibited the effect of *E. fredii* LP2/20 immobilized in agar on the soil bacterial community structure. The proportions of the alpha-, beta-, gamma-, and delta-*Proteobacteria*, unidentified *Cyanobacteria*, *Acidobacteria* subgroup 6, *Bacilli*, and SCG were altered in response to the *E. fredii* LP2/20 establishment. Soil total P, K, and Ca and soil pH were the dominant factors influencing the soil bacterial community structure.

## Supplementary information

Supplementary Figures.

## Data Availability

All data generated or analyzed during this study has been included in this article. Sequence data has been deposited in the National Center for Biotechnology Information under genomic accession number KU866444 and BioProject accession number SRP154296.
