# Supplementary material for: Inoculation of Ensifer fredii strain LP2/20 immobilized in agar results in growth promotion and alteration of bacterial community structure of Chinese kale planted soil
Source: Sci Rep. 2020 Sep 28;10:15857. doi: 10.1038/s41598-020-72986-5 (PMC7522984; doi:10.1038/s41598-020-72986-5)
Supplement: Supplementary file 1 — Supplementary Figures. [file 41598_2020_72986_MOESM1_ESM.docx]

**SUPPLEMENTARY INFORMATION**

# Inoculation of *Ensifer fredii* strain LP2/20 immobilized in agar results in growth promotion and alteration of bacterial community structure of Chinese kale planted soil

# Neelawan Pongsilp^1^ and Pongrawee Nimnoi^2^*

# ^1^ Department of Microbiology, Faculty of Science, Silpakorn University, Nakhon Pathom 7300, Thailand

# ^2^ Department of Microbiology, Faculty of Liberal Arts and Science, Kasetsart University, Nakhon Pathom 73140, Thailand

# *Corresponding author: Pongrawee Nimnoi

# Email: umco_perra@hotmail.com; faaspwn@ku.ac.th

**SUPPLEMENTARY FIGURE**

**
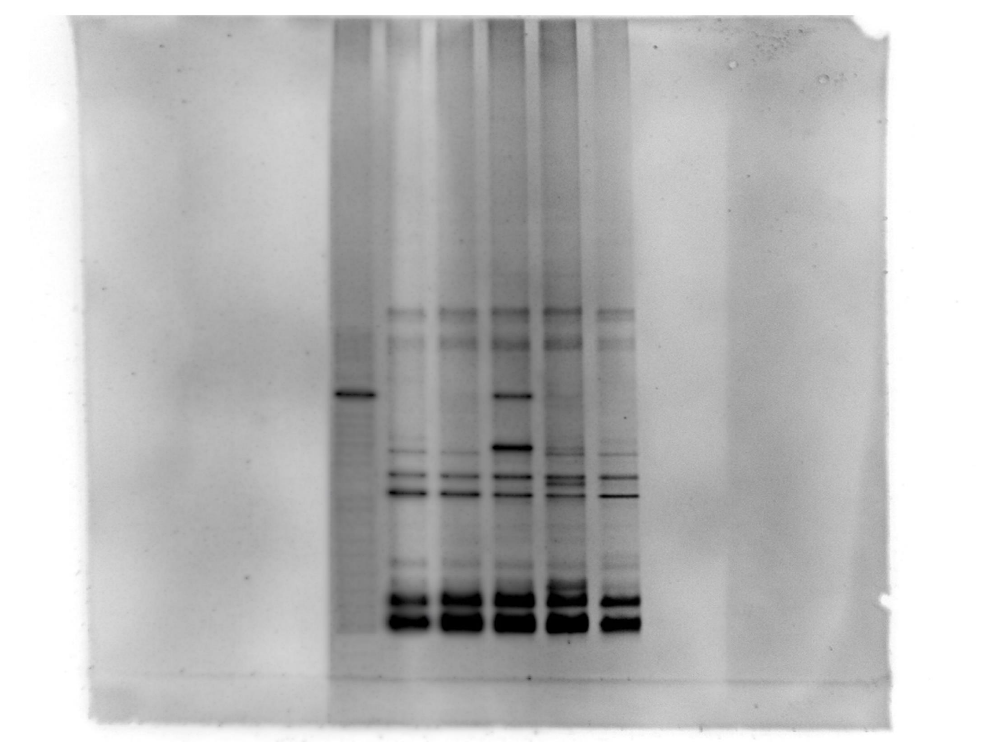
**

**Supplementary Figure S1. The original picture of RT-PCR-DGGE fingerprintings of soil from five treatments at 50 days after planting (replicate 1).** Lane 1 (start from left hand side), reference band of the strain; Lane 2, uninoculated control; Lane 3, inoculation with the strain in liquid medium; Lane 4, inoculation with the strain immobilized in agar; Lane 5, inoculation with the strain immobilized in alginate, Lane 6, inoculation with the strain immobilized in perlite.


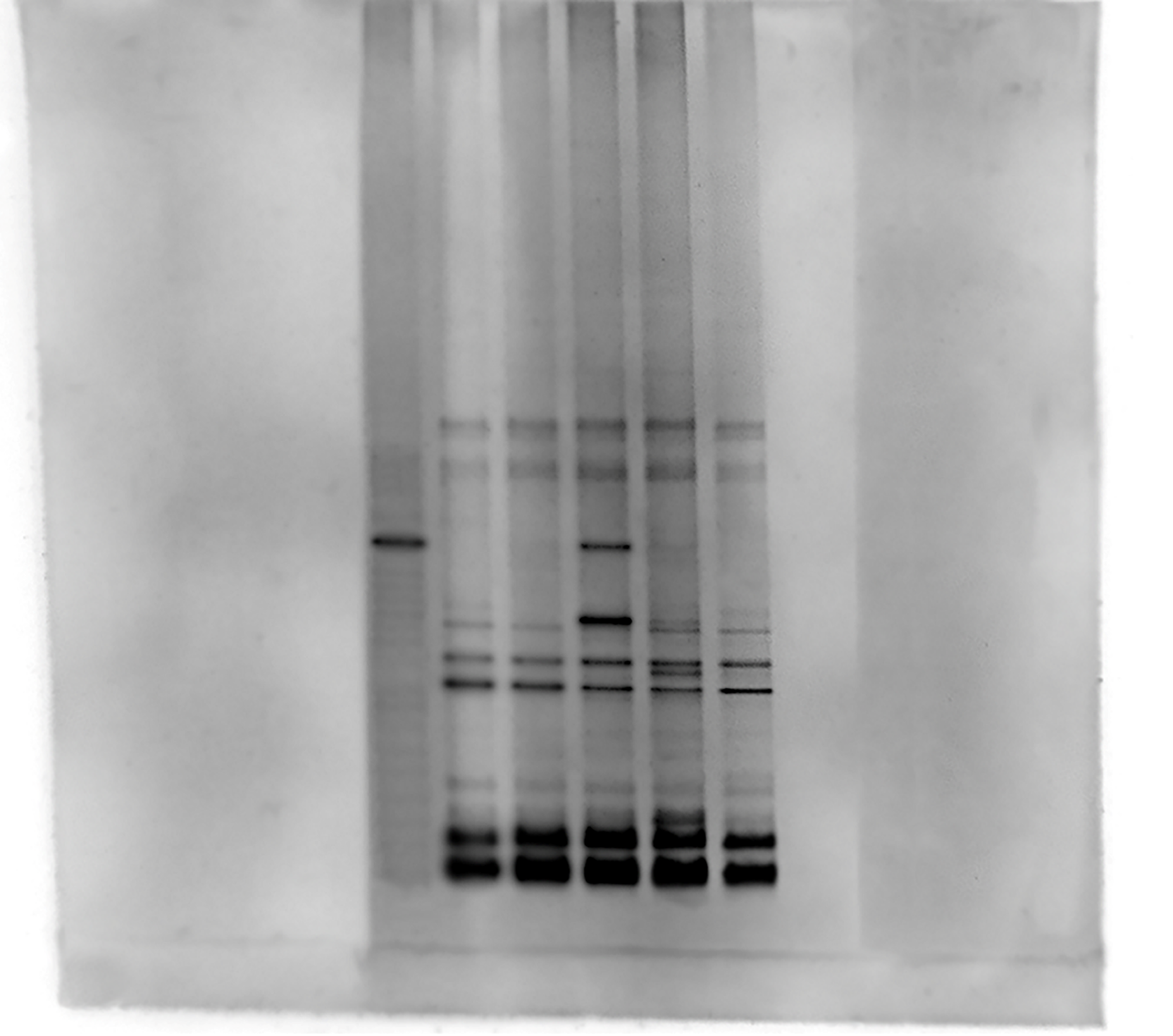


**Supplementary Figure S2. The original picture of RT-PCR-DGGE fingerprintings of soil from five treatments at 50 days after planting (replicate 2).** Lane 1 (start from left hand side), reference band of the strain; Lane 2, uninoculated control; Lane 3, inoculation with the strain in liquid medium; Lane 4, inoculation with the strain immobilized in agar; Lane 5, inoculation with the strain immobilized in alginate, Lane 6, inoculation with the strain immobilized in perlite.


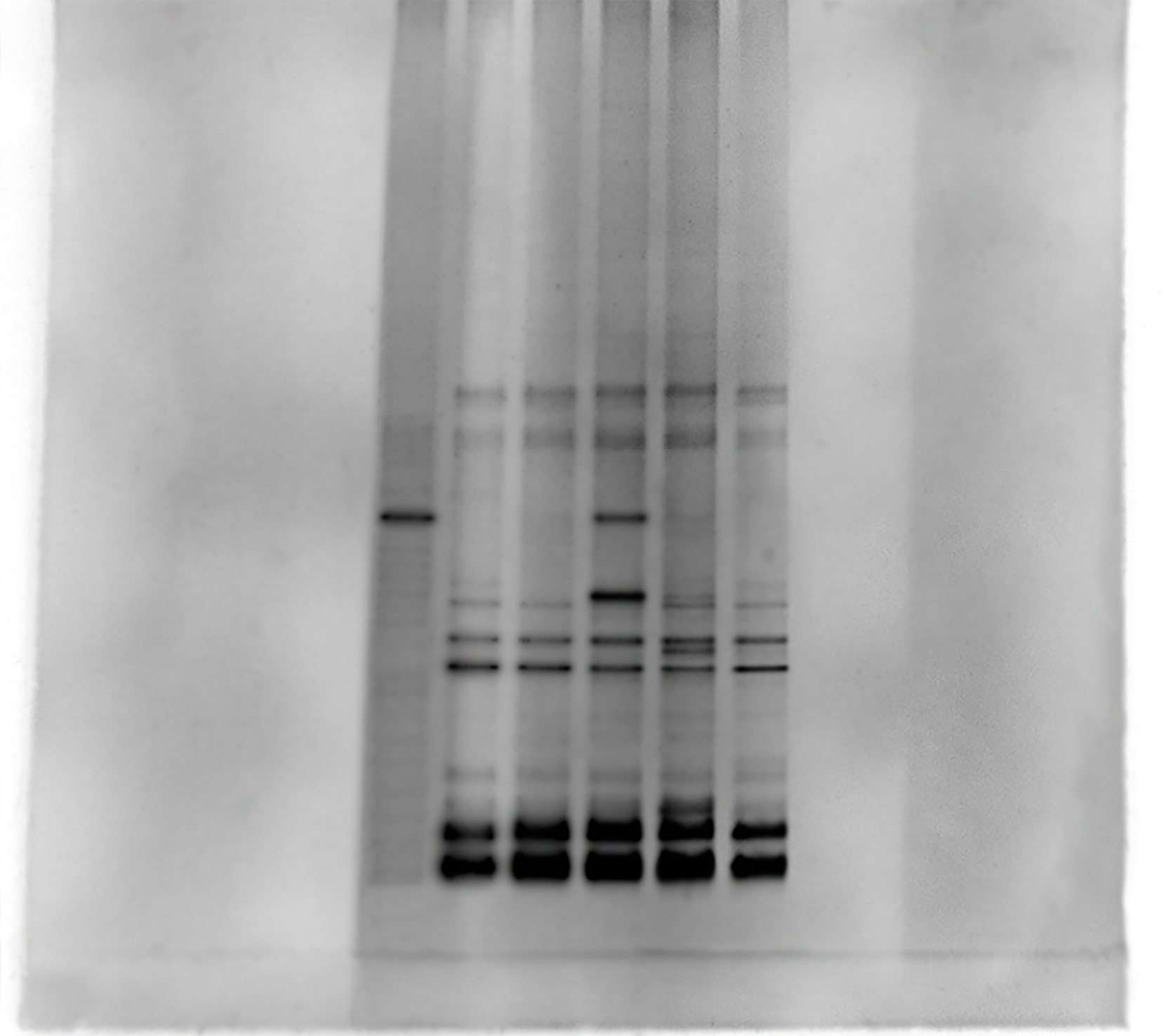


**Supplementary Figure S3. The original picture of RT-PCR-DGGE fingerprintings of soil from five treatments at 50 days after planting (replicate 3).** Lane 1 (start from left hand side), reference band of the strain; Lane 2, uninoculated control; Lane 3, inoculation with the strain in liquid medium; Lane 4, inoculation with the strain immobilized in agar; Lane 5, inoculation with the strain immobilized in alginate, Lane 6, inoculation with the strain immobilized in perlite.
